# Climate Classification is an Important Factor in Assessing Quality-of-Care Across Hospitals

**DOI:** 10.1038/s41598-017-04708-3

**Published:** 2017-07-10

**Authors:** Mary Regina Boland, Pradipta Parhi, Pierre Gentine, Nicholas P. Tatonetti

**Affiliations:** 1 Department of Biomedical Informatics, Columbia University, New York, NY USA; 2 Department of Systems Biology, Columbia University, New York, NY USA; 3 Department of Medicine, Columbia University, New York, NY USA; 4 Observational Health Data Sciences and Informatics, Columbia University, New York, NY USA; 5 Department of Earth and Environmental Engineering, Columbia University, New York, NY USA

## Abstract

Climate is a known modulator of disease, but its impact on hospital performance metrics remains unstudied. We assess the relationship between Köppen-Geiger climate classification and hospital performance metrics, specifically 30-day mortality, as reported in Hospital Compare, and collected for the period July 2013 through June 2014 (7/1/2013–06/30/2014). A hospital-level multivariate linear regression analysis was performed while controlling for known socioeconomic factors to explore the relationship between all-cause mortality and climate. Hospital performance scores were obtained from 4,524 hospitals belonging to 15 distinct Köppen-Geiger climates and 2,373 unique counties. Model results revealed that hospital performance metrics for mortality showed significant climate dependence (p < 0.001) after adjusting for socioeconomic factors. Climate is a significant factor in evaluating hospital 30-day mortality rates. These results demonstrate that climate classification is an important factor when comparing hospital performance across the United States.

## Introduction

The relationship between climate and human health and disease is well known^1–5^. Recently, variation in temperature related to climate variability and extreme weather events has been linked to hospital admission rates for heart disease^6^. Air humidity is also an important health factor^7^. Pollutants, including both carbon monoxide and fine air particulates have been associated with increased admissions for a number of conditions^8, 9^. Outcome-based quality of care assessments ignore hospital location when comparing hospitals and explicitly state the assumption that ‘global location of the hospital should not affect the outcome’^10^ despite the evidence to the contrary^11^.

Hospital performance statistics are often reported while ignoring regional climate information. Location is usually only mentioned as a confounding factor in determining the fiscal cost of services^12^ while ignoring the quality of the result (i.e., the 30-day mortality rate). Many climate variables (e.g., temperature, humidity) and other climate-dependent pollutants (e.g., air particulates, water contaminants) may be responsible for some of the variation in hospital performance metrics. The total number of relevant climate variables to consider can be large. Therefore, we selected the Köppen-Geiger climate classification system^13, 14^ as a proxy for a high number of intertwined climate variables. The Köppen-Geiger climate classification uses precipitation and temperature to describe a region’s climate and as been widely used in various fields such as hydrology^15^ and ecology^16^. Climate classification is an important variable when studying health-related effects as climate determines many facets of season. For example, some climates only experience two principle seasons (e.g., wet vs dry season) while others experience the more traditional ‘four season’ climate. Therefore, we selected the Köppen-Geiger climate classification system, which models various facets of climate that also affect season intensities. In addition to climate, adjusting for socioeconomic factors is important when studying climate effects as prior studies have shown significant geographic gradients in hospital services that are economic-dependent and not climate-dependent^17^.

Hospital performance statistics and 30-day mortality rates for many hospitals across the United States of America (USA) are readily available in the Hospital Compare dataset. Hospital Compare is maintained by the Centers for Medicare and Medicaid Services (CMS) with the purpose of providing the general public with information to make informed decisions on their healthcare (www.hospitalcompare.hhs.gov).

In this study, we examined hospital-level variation in 30-day mortality measures reported in Hospital Compare and their relationship with hospitals’ Köppen-Geiger climate classification while adjusting for socioeconomic confounders. Our study uses only publically available datasets.

## Methods

### Data

#### Köppen-Geiger climate classification

We used the Köppen-Geiger climate classification system^13, 18^ to compare hospitals’ climates. The Köppen-Geiger classification is a classical climate classification used by researchers^19^ throughout the world including members of the World Health Organization^20^. Each county in the United States of America (USA) is assigned a category using three axes: broad climate type, as well as precipitation and temperature characteristics^13, 14^ (SI Appendix Table S1). Each of these three factors is combined to produce the overall climate designation. For example, the New York City (NYC) climate is designated Cfa meaning that its climate is warm temperate (‘C’), fully humid (‘f’) with a hot summer (‘a’). We used a Köppen-Geiger climate at the US-county level^14^ and linked it to Federal Information Processing Standard (FIPS) codes. In some instances, multiple climate classes were mapped to the same county with proportions for each climate within that county (this occurs when counties contain data from across two climate boundaries). Therefore, we took the climate with the highest proportion for each county (i.e., the dominant climate) for our analysis.

#### Hospital Compare

We obtained 30-day mortality data from Hospital Compare by downloading the entire release of Hospital Compare (2015 Annual Files released on July 16, 2015)^21^. We used the ‘readmissions, complications and deaths’ file to obtain 30-day mortality rates for all conditions reported (a total of 6 conditions, obtained from file: HOSArchive_Revised_FlatFiles_20150716/Readmissions and Deaths-Hospital.csv). Data for these measures were collected for the period from July 2011 through June 2014 (7/1/2011–6/30/2014). We did not investigate patient-reported outcomes^22^. Each hospital included in Hospital Compare contains zip code information, which can be used to link the hospital to the county-level Köppen-Geiger climate data source (described above).

#### Census Bureau’s American Community Survey (ACS)

Socioeconomic factors are known confounders in quality-of-care comparisons across hospitals^23^. Therefore, we obtained data on six different potentially confounding variables for hospital performance: income, race, English-speaking ability, insurance coverage, renter-occupied status, and total number of households. We used the American Community Survey (ACS) collected by the U.S. Census Bureau, 5-year data from the 2014 release. Additional details on extraction process are contained in SI Appendix.

We selected these six potential confounders because they have been linked to hospital performance (either readmission or mortality) metrics and are known to vary regionally. Socioeconomic status (specifically median household income and race) are well-known independent risk factors for hospital readmissions related to heart failure^24^. Patients who owned their own homes had significantly fewer hospital readmissions for COPD^25^ indicating that the percent of renters per county is a potential confounder. English fluency is another potential confounder as non-English speaking individuals were at an increased risk for 30-day readmissions even after adjusting for other socioeconomic variables^26^. Insurance status also altered risk for hospital readmissions^27^.

### Statistical Methods

We extracted all six mortality metrics for each hospital provided by Hospital Compare. Each score was reported in percentages. For example, a hospital score of 14.6 for’Heart Failure (HF) 30-Day Mortality Rate” indicates that 14.6% of patients with a heart failure diagnosis died within 30-days. We used the raw mortality counts along with the counts of patients sampled for each hospital instead of the raw mortality rates. We mapped the Hospital Compare data to their corresponding FIPS codes using the zip codes provided in the original data file using the R package noncensus^28^.

We mapped the Köppen-Geiger climate classes to the Hospital Compare data. We removed all scores that were reported as ‘Not Available’ by the CMS. The CMS lists data as ‘Not Available’ if 1) the number of cases does not meet the minimum required for public reporting, 2) the number of cases is too small to reliability report the data, or 3) to protect personal health information. The CMS may also restrict access to mortality rates if 1) a hospital elected not to submit data for a particular reporting period, 2) a hospital had no claims data for a particular measure or 3) a hospital elected to suppress a measure from being publicly reported. Since the CMS chose to restrict these data, they were unavailable to us for analyses. We also removed four hospitals belonging to a unique climate (i.e., with no other hospital in that climate) to avoid any biases due to low-sample size. Our final dataset contained 4,524 hospitals belonging to 15 distinct climates. We used an F-test to determine overall significance for the various measures and confounders vs. climate.

We constructed a linear hierarchical regression model that pools data from all six 30-day mortality rates reported in Hospital Compare. This model was implemented using the Stan software^29^ in R version 3.3.0 (2016-05-03)^30^. The model then automatically captures the relationship between size of the hospital and natural sampling fluctuations. We fitted the model using the open-source Bayesian inference engine, running at the default settings of 2000 iterations for 4 chains. Parameters for hospital size, and hospital-specific variability in mortality were used in the model as these factors could skew the results^31^. In addition, parameters for each of the six-socioeconomic confounder variables were used. These socioeconomic factors included: income, total number of households, % renter, % un-insured, % speak English ‘very well’, and % white alone. Data for each socioeconomic variable were obtained from the ACS using the county-level FIPS codes. Therefore, hospitals were assigned their corresponding socioeconomic variables based on the geographic location of the hospital (i.e., all hospitals in the same county received the same set of socioeconomic variables).

## Results

**Figure 1:**
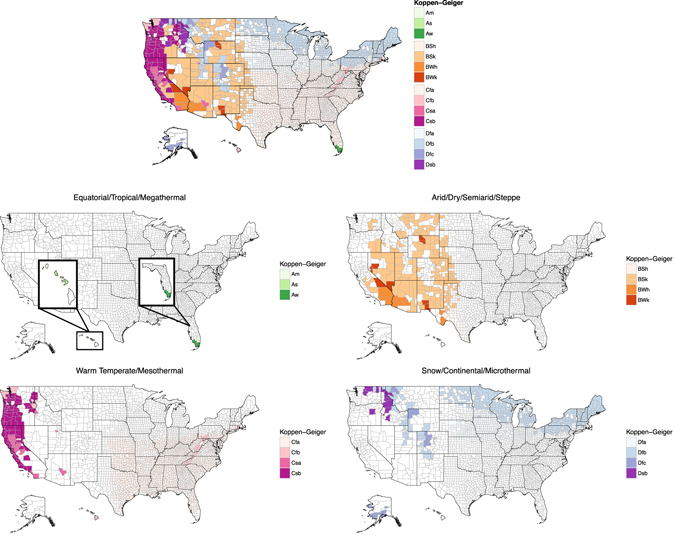
Hospital Compare Data By County with Major Climate Designations: A Map of the United States Showing Hospital Compare Data Mapped to Köppen-Geiger Climate Classifications. Map of the United States was generated in R^30^, using the following libraries: choroplethr (version: ‘3.5.2’, https://cran.r-project.org/web/packages/choroplethr/index.html), ggplot2 (version: ‘2.1.0’, https://cran.r-project.org/web/packages/ggplot2/index.html), noncensus (version: ‘0.1’, https://cran.r-project.org/web/packages/noncensus/index.html), zipcode (version: ‘1.0’, https://cran.r-project.org/web/packages/zipcode/index.html), grid (version: ‘3.3.0’, https://stat.ethz.ch/R-manual/R-devel/library/grid/html/00Index.html) and gridExtra (version: ‘2.2.1’, https://cran.r-project.org/web/packages/gridExtra/index.html). The map itself utilized the choroplethr library version 3.5.2.

Our final dataset contained 4,524 hospitals from 15 distinct climates and 2,373 unique counties (Fig. 1). The state-breakdown for the number of counties represented in our sample versus the total number of counties is provided (SI Appendix Table S2). We also reported the six mortality measures varied by climate (Table S3, and graphically illustrated in SI Figure S1 **)**. The six socioeconomic confounder variables also varied across climates (Table S4 **)** and the counties in the USA (Figure S2 **)**. Each confounder was significant across different climates, which motivated us to adjust for these socioeconomic confounders in our model.

**Figure 2:**
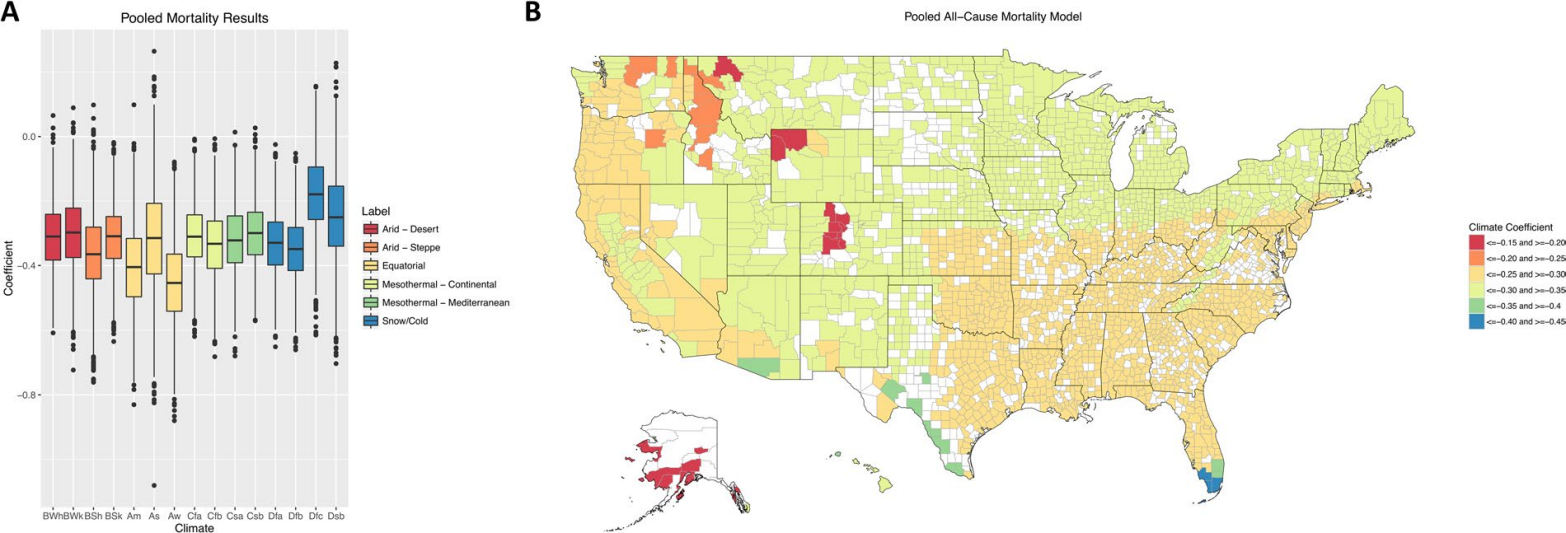
Climate’s Impact on Hospital Performance Mortality Statistics After Adjustment for Confounders: Map of the United States of America. Model coefficients for climate’s impact on 30-day mortality are displayed by climate classification in Fig. 2A. A map of the USA illustrating the results of the model is shown in Fig. 2B. Map of the United States was generated in R^30^, using the following libraries: choroplethr (version: ‘3.5.2’, https://cran.r-project.org/web/packages/choroplethr/index.html), and ggplot2 (version: ‘2.1.0’, https://cran.r-project.org/web/packages/ggplot2/index.html). The map itself utilized the choroplethr library version 3.5.2.

Our linear regression model revealed distinct relationships between climate and mortality statistics. We pooled the mortality data across all six conditions and found that equatorial climates (light orange Fig. 2A) had lower climate coefficients after adjustment for confounding. Hospitals located in equatorial dry climates (Aw climate) were found with the lowest risk of mortality (blue in Fig. 2B) indicating that wetter climates are more debilitating a finding also reported by Sherwood *et al*.’s model^7^. The subarctic snow climate (Dfc climate) had the highest pooled mortality rate (red in Fig. 2B). Another climate with high mortality is the snowy warm summer climate (orange in Fig. 2B, Dsb climate). Both the subarctic snow climate (Dfc) and the snowy warm summer climate (Dsb) are centered in the northwest region of the USA.

## Discussion

Our results suggest the importance of considering climate-induced impact in hospital performance statistics. We found that pooled 30-day mortality rates after discharge varied significantly by climate. In general, equatorial climates had improved mortality rates after adjusting for socioeconomic confounders. Further, subarctic and heavy snow climates had worse mortality rates. We adjusted for known confounders, including, % insured, % renters, household income, % speak English well, % white and total number of households. We found a significant climate effect after adjusting for these factors. Our results fit well with the literature. For example, cold, dry air (i.e., low temperature and low humidity) is known to increase the risk of influenza-related mortality^32, 33^ and we found that colder climates tended to have higher mortality rates while warmer, milder climates had lower mortality rates. Additionally, Sherwood *et al*. describe how habitable regions of the earth never exceed 31 C and an excess of 35 C could induce hyperthermia^7^. These temperatures are extremes and the corresponding phenotypes are equally extreme (i.e., death). They posit that trends in the mammalian fossil record may be due to extreme variation in temperature that affected habitability^7^. However, it is likely that even smaller variations in temperature, as observed in different climates, is likely to affect human health although to a lesser extent.

### Policy Implications Of Climate – Performance Relationship

In the United States of America, various federal agencies, such as CMS, determine the allocation of funds to hospitals. Previously, CMS paid hospitals based on the volume of Medicare or Medicaid recipients who received services at a particular hospital. Patients enrolled in Medicare could receive care at any hospital, and the federal government (through the CMS) allocated payments based on the service provided and the volume per hospital. Therefore, a patient could receive care at a hospital outside of their county of residence, assuming the patient had the resources to travel to that hospital.

The new CMS reimbursement model incorporates information on hospital quality into the fund allocation model. This is called ‘Hospital Value-Based Purchasing’ the total performance score of a hospital is decomposed into sections with 25% of the score being determined by clinical care outcomes (such as mortality) for fiscal year 2017^34, 35^. We have focused on mortality in this study given the importance of mortality in both reimbursement calculations and also to patients who are interested in improving their outcomes. Tremendous strides have been made to provide more data to patients to allow patients to make informed healthcare choices. The CMS designed Hospital Compare as a patient-facing tool to allow patients to ‘compare’ hospitals using a set of hospital performance metrics. This allows patients to more easily decide where they want to receive medical treatment.

Some researchers have questioned the usefulness of Hospital Compare’s performance metrics^36^. They note a discrepancy between “Hospital Compare” quality measures and “Best Hospitals”^36^. However, researchers have shown that the reported conditions (heart attack, pneumonia, heart failure) that Hospital Compare highlights account for 16% of Medicare discharges from acute care hospitals and 16% of Medicare hospital payments^37^. This makes the Hospital Compare performance metrics vital for a large cohort of Medicare patients.

Additionally, alternative metrics such as “Best Hospitals” have not proven to be informative in predicting outcomes such as 30-day mortality. One study investigated surgical outcomes following radical cystectomy found either no correlation or an inverse correlation between the quality of the hospital (using one of these “Best Hospitals” metrics) and mortality 90 days following surgery^38^ making those metrics not informative for measuring mortality-related hospital performance. Others found that admission to one of “America’s Best Hospitals” was associated with lower 30-day mortality among elderly patients with acute myocardial infarction^39^. Although, those researchers did not mention adjusting for other socioeconomic confounders (e.g., insurance status) that would bias their results^39^.

Hospital Compare performance measures have been used successfully by many researchers to learn more about hospital care through the United States^40^. Using these data, we demonstrate that climate matters when choosing a hospital even after adjusting for many other known socio-economic factors (Fig. 2B). These results have important implications for patients as well as policy makers.

### Proposed Climate-Based Performance Adjustment

Originally, hospitals’ mortality rates were compared against a national rate. However, due to unfair penalization of hospitals in poor-regions, adjustments were made for socioeconomic status^41^. In this study, we found that climate is another factor in hospital mortality rates even after adjusting for socioeconomic status. In other words, patients in certain climates experience a higher degree of mortality that is independent of their income. Therefore, we propose that financial adjustments should be made for hospitals based on their performance relative to other hospitals within the same climate. This would allow hospitals to be meaningfully compared to each other. Climate-based adjustments should be considered by policy makers to make meaningful comparisons across different hospitals. Otherwise, hospitals in more extreme climates will be unfairly penalized. The higher mortality observed in those locations is not due to lower quality of care, but more harsh climate conditions.

### Limitations

There are some limitations to our work including our exclusive use of Hospital Compare’s 30-day mortality metrics. These metrics do not fully represent the quality of a given hospital^42^. However, these metrics are used to assess hospital quality by policy makers and these mortality measures represent a significant burden on the Medicare population^37^. We used 30-day mortality rates, as these were readily available from Hospital Compare. However, some researchers suggest that shorter time intervals may be more conducive to quality-of-care assessments^43^, however these shorter time intervals are not currently being provided publically. Another factor of importance in mortality (but not included in hospital reimbursement models) is pollution. Climate factors can affect individuals’ exposure to pollution and the modes of exposure. For example, heavy rainfall purifies the air of pollutants while contaminating the water. Therefore, pollution and its deadly effects will also vary by climate. We focus our work on the effect of climate on hospital performance mortality metrics while adjusting for socioeconomic confounders. Some of the climate effects we observe could be masked effects of pollution or some other unknown climate-based exposure.

## Conclusion

Our study demonstrates that climate-induced impact on hospital performance metrics exists. This climate-based variation in mortality rates exists among hospitals even after adjusting for socioeconomic confounders. Our findings are important for policy makers as climate-based adjustments for hospitals could be conducted to enable ‘meaningful comparisons’ of hospitals by comparing hospitals within the same climate. Additionally, our findings are important for researchers that study hospital performance as the Köppen-Geiger climate represents an important, yet often overlooked, variable in the equation.

## Electronic supplementary material

Supplemental Information
