# Supplementary material for: Climate Classification is an Important Factor in Assessing Quality-of-Care Across Hospitals
Source: Sci Rep. 2017 Jul 10;7:4948. doi: 10.1038/s41598-017-04708-3 (PMC5504082; doi:10.1038/s41598-017-04708-3)
Supplement: Supplementary file 1 — Supplemental Information [file 41598_2017_4708_MOESM1_ESM.doc]

**Supplemental Appendix for:**

**Climate Classification is an Important Factor in Assessing Quality-of-Care Across Hospitals**

**Boland MR, Parhi P, Gentine P, Tatonetti NP.**

**Table of Contents**

*Additional Details On Methods*

Notes on Obtaining Data from Census Bureau’s American Community Survey (ACS)…………………..……..2

*Figures*

Figure S1. Raw Mortality Boxplots For All 6 Mortality Measures By Köppen-Geiger Climate Classification System…………………….…..…………………………………………………………………………………...3

Figure S2. County-Level Variance of Six Known Confounders: income, total number of households, % renter occupied housing, % uninsured persons, % English-fluent and % white. ……………………………...………...4

*Tables*

Table S1. Köppen-Geiger Three Tiered Climate Classification System…………………….…..………………..6

Table S2. Percent of Counties Represented in Hospital Compare Sample: State Breakdown…..………………..7

Table S3. Distribution of 6 Mortality Measures Across 15 Climates Included in Study…………………………9

Table S4. Distribution of Confounding Variables Across All 19 Climates For Hospitals With Data.…...……..10

**Notes on Obtaining Data from Census Bureau’s American Community Survey (ACS)**

We obtained data on six different potentially confounding variables for hospital performance. We used the American Community Survey (ACS) collected by the U.S. Census Bureau, 5-year data from the 2014 release. Fact Finder was used to download the relevant data tables from the ACS website. We obtained median household income and total number of households from table S1903(1) ; level of English speaking ability from Table S1601 (2); health insurance coverage status from Table S2701(3); race from Table B02008 (4); and renter-occupied status from Table S2502 (5). All data were obtained at the county-level and contained FIPS codes for linkage with climate and hospital data.

**Figure S1. Raw Mortality Boxplots For All Six Mortality Measures By Köppen-Geiger Climate Classification System.** The relationship between climate and individual disease varies somewhat. A near linear relationship is observed for 30-day heart failure mortality (lower center plot). A pooled-mortality statistic (across all 6 diseases) was used in the model. Köppen-Geiger Model data obtained from (6, 7). Plot implemented in R (8).

**Figure S2. County-Level Variance of Six Known Confounders: income, total number of households, % renter occupied housing, % uninsured persons, % English-fluent and % white.** Map of the United States was generated in R (8) using the following libraries: choroplethr (version: ‘3.5.2’, URL: https://cran.r-project.org/web/packages/choroplethr/index.html), ggplot2 (version: ‘2.1.0’, URL: https://cran.r-project.org/web/packages/ggplot2/index.html), noncensus (version: ‘0.1’, URL: https://cran.r-project.org/web/packages/noncensus/index.html), zipcode (version: ‘1.0’, URL: https://cran.r-project.org/web/packages/zipcode/index.html), grid (version: ‘3.3.0’, URL: <https://stat.ethz.ch/R-manual/R-devel/library/grid/html/00Index.html>) and gridExtra (version: ‘2.2.1’, URL: https://cran.r-project.org/web/packages/gridExtra/index.html). The map itself utilized the choroplethr library version 3.5.2.

**Table S1. Köppen**-Geiger Three Tiered Climate Classification System

| **Main Climate** | **Precipitation** | **Temperature** |
| --- | --- | --- |
| A: equatorial | W: desert | h: hot arid |
| B: arid | S: steppe | k: cold arid |
| C: warm temperate | f: fully humid | a: hot summer |
| D: snow | s: summer dry | b: warm summer |
| E: polar | w: winter dry | c: cool summer |
|  | m: monsoonal | d: extremely continental |
|  |  | F: polar frost |
|  |  | T: polar tundra |

Köppen-Geiger Model data obtained from (6, 7).

**Table S2. Percent of Counties Represented in Hospital Compare Sample: State Breakdown**

| **State** | **Total No. Of Counties In Hospital Compare Sample Used In Study *** | **Total No. Of Counties** | **% Counties Represented** |
| --- | --- | --- | --- |
| AK | 11 | 29 | 37.9 |
| AL | 57 | 67 | 85.1 |
| AR | 53 | 76 | 69.7 |
| AZ | 14 | 15 | 93.3 |
| CA | 56 | 58 | 96.6 |
| CO | 46 | 64 | 71.9 |
| CT | 8 | 8 | 100 |
| DC | 1 | 1 | 100 |
| DE | 3 | 3 | 100 |
| FL | 56 | 67 | 83.6 |
| GA | 103 | 160 | 64.4 |
| HI | 4 | 5 | 80 |
| IA | 84 | 100 | 84 |
| ID | 31 | 44 | 70.5 |
| IL | 76 | 103 | 73.8 |
| IN | 76 | 92 | 82.6 |
| KS | 89 | 105 | 84.8 |
| KY | 79 | 120 | 65.8 |
| LA | 53 | 64 | 82.8 |
| MA | 14 | 14 | 100 |
| MD | 20 | 25 | 80 |
| ME | 15 | 16 | 93.8 |
| MI | 68 | 83 | 81.9 |
| MN | 73 | 87 | 83.9 |
| MO | 66 | 116 | 56.9 |
| MS | 69 | 82 | 84.1 |
| MT | 38 | 56 | 67.9 |
| NC | 81 | 100 | 81 |
| ND | 30 | 53 | 56.6 |
| NE | 65 | 93 | 69.9 |
| NH | 10 | 10 | 100 |
| NJ | 21 | 21 | 100 |
| NM | 26 | 33 | 78.8 |
| NV | 12 | 17 | 70.6 |
| NY | 56 | 62 | 90.3 |
| OH | 79 | 88 | 89.8 |
| OK | 73 | 77 | 94.8 |
| OR | 31 | 37 | 83.8 |
| PA | 60 | 67 | 89.6 |
| RI | 4 | 5 | 80 |
| SC | 39 | 46 | 84.8 |
| SD | 35 | 67 | 52.2 |
| TN | 75 | 96 | 78.1 |
| TX | 162 | 255 | 63.5 |
| UT | 23 | 29 | 79.3 |
| VA | 61 | 133 | 45.9 |
| VT | 12 | 14 | 85.7 |
| WA | 34 | 39 | 87.2 |
| WI | 63 | 72 | 87.5 |
| WV | 41 | 55 | 74.5 |
| WY | 21 | 23 | 91.3 |

*** This is the Number of Counties For Hospitals Included In Study (i.e., Excludes The 4 Hospitals Removed Because They Were The Only Representatives Of Their Climate)**

**Table S3. Distribution of 6 Mortality Measures of Hospital Performance**

**Across 15 Climates Included in Study**

| **Climate (example city)** | Acute Myocardial Infarction (AMI) 30-Day Mortality Rate * | Death rate for CABG * | Death rate for chronic obstructive pulmonary disease (COPD) patients * | Heart failure (HF) 30-Day Mortality Rate * | Pneumonia (PN) 30-Day Mortality Rate * | Death rate for stroke patients * |
| --- | --- | --- | --- | --- | --- | --- |
| Cfa (New York City, NY) | 14.2 ± 1.3 | 3.4 ± 0.9 | 7.7 ± 1 | 11.6 ± 1.4 | 11.8 ± 1.8 | 14.7 ± 1.6 |
| Dfb (Portland, ME) | 14.1 ± 1.2 | 3 ± 0.7 | 7.8 ± 1 | 11.9 ± 1.4 | 11.4 ± 1.6 | 15.1 ± 1.6 |
| Dfa (Chicago, IL) | 14 ± 1.2 | 3.2 ± 0.8 | 7.7 ± 1 | 11.7 ± 1.6 | 11.6 ± 1.7 | 14.7 ± 1.7 |
| BSk (Billings, MT) | 14.2 ± 1.3 | 3.6 ± 0.8 | 8.1 ± 1 | 12 ± 1.2 | 11.7 ± 1.4 | 15.6 ± 1.4 |
| Csb (Carson City, NV) | 14 ± 1.2 | 3.1 ± 0.6 | 7.8 ± 1.2 | 11.7 ± 1.8 | 11.3 ± 1.9 | 14.8 ± 1.9 |
| Csa (Ione, CA) | 13.9 ± 1.1 | 3 ± 0.7 | 8 ± 1.4 | 11.5 ± 1.5 | 11.4 ± 1.7 | 14.7 ± 1.6 |
| Cfb (Kamiah, ID) | 14 ± 1.2 | 3.2 ± 0.8 | 8 ± 1.1 | 11.8 ± 1.3 | 11.6 ± 1.3 | 15 ± 1.5 |
| BWh (Monahans, TX) | 14.1 ± 1.1 | 3.1 ± 0.6 | 7.8 ± 1 | 11.2 ± 1 | 11.3 ± 1.7 | 14.5 ± 1.6 |
| BWk (Fallon, NV) | 15 ± 1.4 | 3.6 ± 0.8 | 8.2 ± 1 | 12.3 ± 1.5 | 12.5 ± 1.7 | 16 ± 1.8 |
| Dfc (Naknek, AK) | 14 ± 1 | 2.9 ± 0.4 | 8.2 ± 1 | 12.5 ± 1.4 | 11.9 ± 1.3 | 15.4 ± 1.6 |
| Am (Fort Lauderdale, FL) | 14.1 ± 1 | 3 ± 0.8 | 7.8 ± 0.8 | 11 ± 1.2 | 10.3 ± 1.3 | 14.6 ± 1.6 |
| BSh (Ozona, TX) | 14.2 ± 1.2 | 3.1 ± 1.3 | 7.7 ± 1.7 | 11.2 ± 1.4 | 10.9 ± 1.6 | 14.4 ± 1.8 |
| Aw (Miami, FL) | 13.7 ± 1.3 | 3 ± 0.6 | 7.6 ± 1 | 11 ± 1.1 | 10.8 ± 1.4 | 14.4 ± 1.5 |
| Dsb (Idaho City, ID) | 14.1 ± NA | 2.8 ± NA | 8.1 ± 1 | 12.7 ± 1.2 | 12.2 ± 1.8 | 15.5 ± 0.9 |
| As (Honolulu, HI) | 14.8 ± 1.1 | 2.9 ± 0.2 | 8.1 ± 1.1 | 11.6 ± 0.9 | 11.1 ± 1.1 | 15.2 ± 1.5 |
| ET (Lake City, CO) ** | Excluded | Excluded | Excluded | Excluded | Excluded | Excluded |
| Dsc (Hailey, ID) ** | Excluded | Excluded | Excluded | Excluded | Excluded | Excluded |
| Dwb (Hettinger, ND) ** | Excluded | Excluded | Excluded | Excluded | Excluded | Excluded |
| Dwa (Martin, SD) ** | Excluded | Excluded | Excluded | Excluded | Excluded | Excluded |
| **F-Stat** | **2.363** | **2.743** | **3.562** | **4.229** | **5.215** | **4.527** |
| **P-value** | **0.003** | **<0.001** | **<0.001** | **<0.001** | **<0.001** | **<0.001** |

Excluded From Statistical Analyses Because There Was Only 1 Hospital In Climate

*mean ± sd

** Removed From Statistical Analyses: Only 1 Hospital In Climate

**Table S4. Distribution of Confounding Variables Across All 19 Climates For Hospitals With Data**

| **Climate** | **No. Of Hospitals** | **% Uninsured*** | **% Renter*** | **% White Only*** | **% Speak English ‘Very Well’*** | **Median Household Income*&** | **Total No. of Households* &** |
| --- | --- | --- | --- | --- | --- | --- | --- |
| Cfa (New York City, NY) | 2129 | 15.1 ± 4.6 | 33.6 ± 10.3 | 74.2 ± 16.9 | 94.8 ± 5.5 | 49.693 ± 15.077 | 166.967 ± 253.118 |
| Dfb (Portland, ME) | 697 | 9.6 ± 3.6 | 29.1 ± 6.7 | 89.5 ± 8.3 | 97.2 ± 2.4 | 52.403 ± 10.762 | 101.03 ± 137.785 |
| Dfa (Chicago, IL) | 672 | 10.9 ± 3.9 | 31.2 ± 8.5 | 82.8 ± 14.4 | 95.1 ± 5 | 52.625 ± 10.471 | 277.724 ± 548.545 |
| Csb (Carson City, NV) | 311 | 15.8 ± 4.1 | 43.8 ± 8.5 | 70.2 ± 15.7 | 85.5 ± 9.1 | 59.399 ± 13.972 | 1026.359 ± 1338.172 |
| BSk (Billings, MT) | 307 | 16.9 ± 4.9 | 33.6 ± 7 | 81.4 ± 14 | 92.1 ± 6.1 | 49.058 ± 10.922 | 73.774 ± 90.632 |
| Csa (Ione, CA) | 86 | 15.1 ± 2.1 | 41.2 ± 6.4 | 69.1 ± 9.9 | 85.2 ± 5.5 | 61.389 ± 11.277 | 596.155 ± 444.29 |
| Cfb (Kamiah, ID) | 80 | 12.9 ± 3.2 | 30.7 ± 7.3 | 86.9 ± 13.3 | 96.3 ± 3.8 | 47.406 ± 12.147 | 67.747 ± 83.785 |
| BWh (Monahans, TX) | 78 | 17.8 ± 1.6 | 37.1 ± 3.6 | 73.1 ± 8.1 | 86.8 ± 5.1 | 53.189 ± 3.973 | 932.647 ± 489.196 |
| BWk (Fallon, NV) | 31 | 21.8 ± 3.3 | 42.4 ± 5.8 | 73.2 ± 10.1 | 83 ± 8.2 | 47.61 ± 5.785 | 480.434 ± 285.639 |
| Am (Fort Lauderdale, FL) | 30 | 20 ± 2.6 | 32.9 ± 2.9 | 67.5 ± 9.5 | 86.2 ± 1.6 | 52.681 ± 2.304 | 575.213 ± 136.969 |
| Dfc (Naknek, AK) | 29 | 18.2 ± 3.9 | 34.2 ± 6.5 | 77.5 ± 21.1 | 95.7 ± 3.9 | 64.824 ± 12.38 | 36.958 ± 37.107 |
| Aw (Miami, FL) | 24 | 27.3 ± 2.2 | 42.8 ± 5.3 | 78 ± 5.1 | 69.8 ± 8.9 | 45.6 ± 5.072 | 684.701 ± 302.435 |
| BSh (Ozona, TX) | 23 | 25.1 ± 9.6 | 35 ± 3.8 | 86.6 ± 6.3 | 78.1 ± 14.9 | 40.953 ± 5.916 | 212.984 ± 151.834 |
| Dsb (Idaho City, ID) | 19 | 18.1 ± 2.5 | 28.3 ± 5.8 | 89.9 ± 6.6 | 96.3 ± 3.9 | 42.379 ± 5.175 | 14.074 ± 8.409 |
| As (Honolulu, HI) | 8 | 6.1 ± 1.3 | 44.8 ± 0.8 | 23.2 ± 4.4 | 86.2 ± 1.6 | 72.385 ± 3.073 | 276.048 ± 87.626 |
| ET (Lake City, CO) ** | 1 | Excluded | Excluded | Excluded | Excluded | Excluded | Excluded |
| Dsc (Hailey, ID) ** | 1 | Excluded | Excluded | Excluded | Excluded | Excluded | Excluded |
| Dwb (Hettinger, ND) ** | 1 | Excluded | Excluded | Excluded | Excluded | Excluded | Excluded |
| Dwa (Martin, SD) ** | 1 | Excluded | Excluded | Excluded | Excluded | Excluded | Excluded |
| **F-Stat** |  | **1146** | **435** | **525** | **1279** | **168** | **88** |
| **P-value** |  | **<0.001** | **<0.001** | **<0.001** | **<0.001** | **<0.001** | **<0.001** |

*mean ± sd

** Removed From Statistical Analyses: Only 1 Hospital In Climate

& indicates in thousands

**References**
